# 3D-engineering of Cellularized Conduits for Peripheral Nerve Regeneration

**DOI:** 10.1038/srep32184

**Published:** 2016-08-30

**Authors:** Yu Hu, Yao Wu, Zhiyuan Gou, Jie Tao, Jiumeng Zhang, Qianqi Liu, Tianyi Kang, Shu Jiang, Siqing Huang, Jiankang He, Shaochen Chen, Yanan Du, Maling Gou

**Affiliations:** 1 State Key Laboratory of Biotherapy and Cancer Center, West China Hospital, Sichuan University, and Collaborative Innovation Center for Biotherapy, Chengdu, Sichuan province, China; 2 Department of Neurosurgery, West China Hospital, Sichuan University, Chengdu, Sichuan province, China; 3 State key laboratory for manufacturing systems engineering, Xi’an Jiaotong University, Xi’an, 710049,China; 4 Department of NanoEngineering, University of California, San Diego, La Jolla, California 92093, USA; 5 Department of Biomedical Engineering, School of Medicine, Tsinghua University, Beijing 100084. China

## Abstract

Tissue engineered conduits have great promise for bridging peripheral nerve defects by providing physical guiding and biological cues. A flexible method for integrating support cells into a conduit with desired architectures is wanted. Here, a 3D-printing technology is adopted to prepare a bio-conduit with designer structures for peripheral nerve regeneration. This bio-conduit is consisted of a cryopolymerized gelatin methacryloyl (cryoGelMA) gel cellularized with adipose-derived stem cells (ASCs). By modeling using 3D-printed “lock and key” moulds, the cryoGelMA gel is structured into conduits with different geometries, such as the designed multichannel or bifurcating and the personalized structures. The cryoGelMA conduit is degradable and could be completely degraded in 2-4 months *in vivo*. The cryoGelMA scaffold supports the attachment, proliferation and survival of the seeded ASCs, and up-regulates the expression of their neurotrophic factors mRNA *in vitro*. After implanted in a rat model, the bio-conduit is capable of supporting the re-innervation across a 10 mm sciatic nerve gap, with results close to that of the autografts in terms of functional and histological assessments. The study describes an indirect 3D-printing technology for fabricating cellularized designer conduits for peripheral nerve regeneration, and could lead to the development of future nerve bio-conduits for clinical use.

Peripheral nerve injury most commonly arises from trauma, and less frequently, secondary tumor resection or congenital defects^1^. Approximately 2–5% of trauma patients experience a peripheral nerve injury and about 100,000 peripheral nerve surgeries are performed each year in the North America^2^. In cases of nerve defects shorter than 5 mm, a direct tension-free neurorrhaphy is the preferred nerve repair procedure^3^. For larger gaps, the current “gold standard” is autologous nerve graft. However, nerve autograft presents a number of disadvantages, including the requirement of a second surgery, donor site morbidity, limited graft availability, size and geometrical mismatch, and the possibility of painful neuroma formation^4^. These limitations have motivated the investigation and development of alternative therapeutic strategies to autograft.

Thus far, many nerve repair strategies, such as nerve guidance scaffolds, physiochemical and biological cues, have been applied to guide and promote nerve regeneration^1^,^4^,^5^. Nerve guidance conduits (NGCs) have been widely used for bridging nerve defects through supporting and directing the sprouting axons, allowing the diffusion/influx of oxygen and nutrients, and preventing the infiltration of inflammatory cells and myofibroblasts^6^. Biomaterials from natural and synthetic polymers have been proposed to construct NGCs for peripheral nerve regeneration^6^. Gelatin, which is essentially denatured collagen, has been commonly used in food, cosmetic industries as well as medicine, such as hemostatic agent and blood volume expander^7^,^8^,^9^. Gelatin scaffolds have also been used extensively for tissue engineering^10^,^11^. For peripheral nerve regeneration, several studies have demonstrated that gelatin conduits are of less cytotoxicity and higher biodegradability, and can promote improved nerve regeneration^8^,^9^,^12^. Gelatin methacryloyl (GelMA) is generated from the modification of gelatin with pendant methacryloyl substitution groups, which confers gelatin the properties of photocrosslinking and radical polymerization^10^. GelMA has suitable biological properties and tunable physical characteristics, and thus has been widely used for biomedical applications, such as engineering of bone, cartilage, and vascular tissues^10^,^13^. The gelatin derivative is an inherently cell-adhesive material comprised of modified natural extracellular matrix (ECM) components that can provide functional cues to the resident cells within the scaffolds and thus can aid in nerve regeneration^14^,^15^. Moreover, cell delivery can be facilitated by priming the support cells *in vitro* before transplanted into the injury site, which reduces the rate of cell loss and leakage to surrounding tissues^11^,^15^.

Peripheral nerve injury may involve one or more nerves with different lengths and injury degrees (ranging from neurapraxia to neurotmesis)^2^,^16^. In addition, the injured nervesmay be varied in geometries^17^. The inherent variance in patient anatomies and injury profiles have motivated the development of personalized treatment for peripheral nerve injury^18^. However, challenges remain for conventional manufacturing methodologies to fabricate nerve conduits with complex or custom geometries, because the conduits are mainly manufactured around cylindrical substrates. The acquired nerve conduits with simpler architectures may not be sufficient to promote structural and functional nerve regeneration^14^,^18^,^19^. Recent advances in materials and manufacturing enable the fabrication of complex or patient-specific devices^20^. Three-dimensional (3D) printing, an additive manufacturing technology, is a powerful tool for fabricating 3D constructs with intricate geometries under computer-aided design/computer aided manufacture (CAD/CAM) system control^21^. The technique currently has been applied to manufacture customized medical devices, such as amputee prosthetics, airway splints, and scaffolds for tissue engineering^22^. However, the current 3D-printed devices are often made from synthetic materials that lack of biological functionality for cell delivery^23^.

The aim of this study was to establish a novel manufacturing method for the fabrication of nerve conduits with designer geometries for peripheral nerve regeneration, using cell-adhesive gelatin cryogels that prepared by cryopolymerization of GelMA (cryoGelMA). CryoGelMA NGCs with complex or custom architectures (multichannel, bifurcating and anatomically accurate) could be manufactured using a commercially available inexpensive desktop 3D printer. Then the degradability and biocompatibility of the cryoGelMA NGCs were evaluated. The introduction of support cells, an important biological cue, into nerve conduits holds great promise for nerve repair by providing a beneficial local microenvironment for the regenerating axons^24^,^25^. Adipose-derived stem cells (ASCs) have attracted considerable attention for nerve tissue engineering, because they are multipotent, and can be easily obtained *via* minimally invasive techniques and be rapidly expanded *in vitro*^26^,^27^. Thus the cryoGelMA NGCs were loaded with ASCs to repair a 10 mm nerve defects in rats, and the ability of promoting nerve regeneration was assessed by walking track analysis, electrophysiological assessment, and histological examination. Our strategy to construct a nerve guidance bio-conduit with designer geometries for peripheral nerve regeneration is outlined in Fig. 1.

## Results

### Fabrication of cryoGelMA NGCs

**Figure 1:**
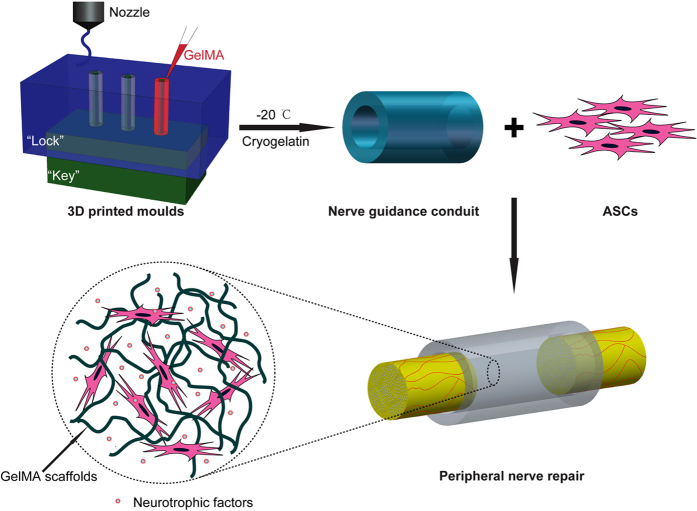
Schematic presentation of the 3D-engineered bio-conduit for peripheral nerve regeneration. Moulds with a “lock and key” structure were fabricated by an indirect 3D printing technique. Then a cryoGelMA NGC was fabricated and seeded with ASCs to bridge a 10 mm sciatic nerve defect.

**Figure 2:**
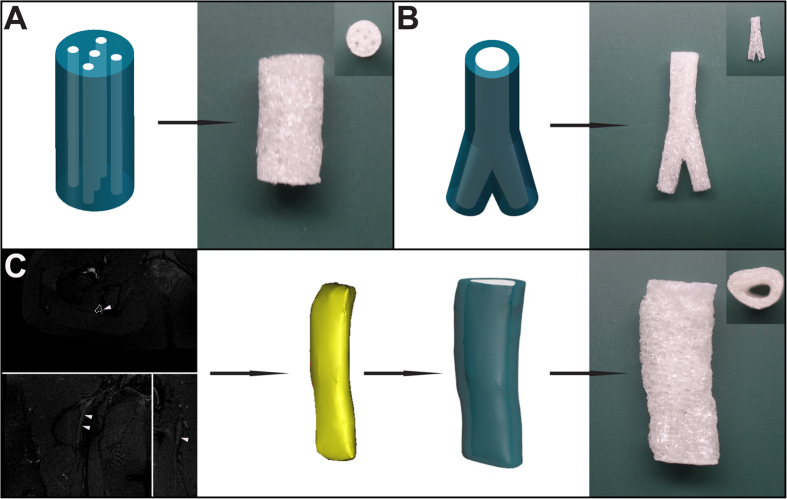
Computer models and photographs of cryoGelMA NGCs with complex geometries, such as multichannel (**A**) and bifurcating (**B**). A patient’s sciatic nerve was reconstructed based on MR neurography, and then a personalized NGC was fabricated (**C**).

Moulds with a “lock and key” structure were fabricated by a 3D printing technique, as shown in Fig. 1. Assisted by the 3D-printed moulds, cryoGelMA NGCs with intricate architectures could be obtained. A multichannel NGC that mimics nerve fascicles and a bifurcating NGC that mimics nerve plexus were successfully constructed (Fig. 2A,B). The appearance of NGCs is white and practically in accordance with the original CAD models. To create a patient-specific NGC that was anatomically accurate, we integrated the indirect 3D printing technique with magnetic resonance (MR) neurography and successfully fabricated a personalized NGC with architectures matching the sciatic nerve of a patient (Fig. 2C). The 3D-printed moulds for molding geometrically complex and patient-specific NGCs are shown in 1A–C.

**Figure 3:**
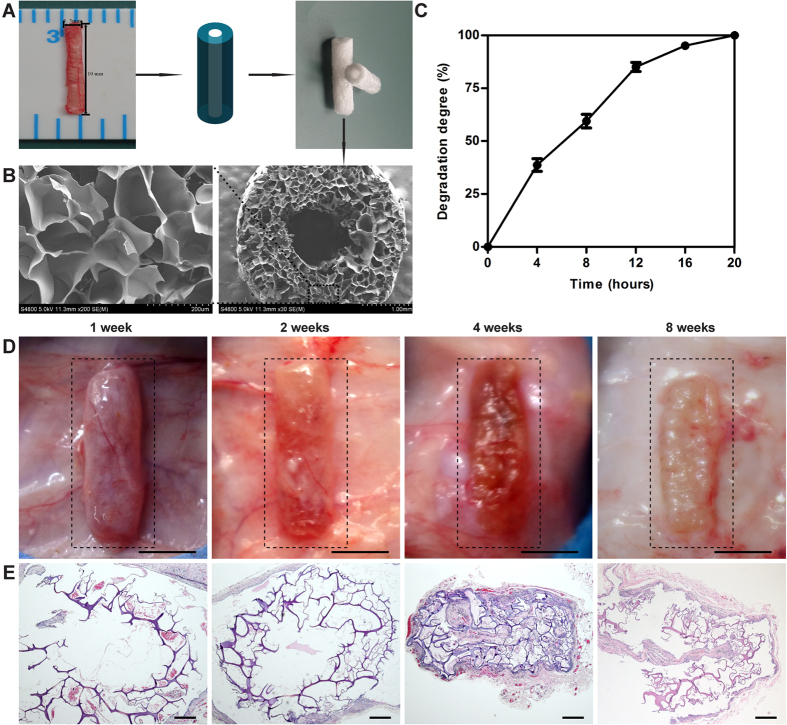
Fabrication, characterization and degradation of cryoGelMA NGCs used for nerve repair in rat sciatic nerve transection model. (**A**) The diameters of the transected sciatic nerve were measured for NGCs design and fabrication. (**B**) SEM micrographs of the NGCs. (**C**) Degradation of the NGCs in presence of collagenase type II (1 mg/ml) solution *in vitro* (n = 3). (**D**) Photographs of the biodegradable NGCs subcutaneously on the dorsal site of rats at 1, 2, 4, and 8 weeks after implantation (scale bars = 5 mm). (**E**) Representative H&E staining of tissue sections of the NGCs at various time point (1, 2, 4, and 8 weeks) after implantation (scale bars = 200 μm).

A cryoGelMA NGC based on the general anatomical features of a rat sciatic nerve was fabricated to test its degradability, biocompatibility, and efficacy in promoting nerve regeneration. A rat sciatic nerve was transected from a Sprague-Dawley (SD) rat weighted 220–250 g to provide a tissue template. A NGC with inner diameter of 1.5 mm, outer diameter of 4.0 mm, and length of 15 mm was fabricated (Fig. 3A). The 3D-printed moulds for molding the NGC are available in Figure S1D. SEM revealed that the NGC had highly porous microstructure that was essential for cell attachment, proliferation, and survival (Fig. 3B). The wall thickness of the fabricated conduits matched with the original CAD model, but the inner diameter was approximately 0.1 mm smaller than the CAD model.

### Degradability of cryoGelMA NGCs

Non-biodegradable conduits are associated with several disadvantages, such as chronic inflammation and a secondary surgery for conduits removal^9^. Thus, biodegradable materials are advocated for the preparation of NGCs. Firstly, we tested whether the cryoGelMA NGCs could be enzymatically degraded *in vitro*. As shown in Fig. 3C, the NGCs could be degraded completely in the presence of 1 mg/ml collagenase type II within 20 h. Then the NGCs were implanted subcutaneously on the dorsal site of rats to evaluate the degradability *in vivo* (Fig. 3D). The NGCs degraded throughout the implantation period, and remained structurally stable for 2 weeks after implantation. The NGCs were collapsed at 4^th^ week, and did not completely degrade in 2 months. The infiltration of fibroblasts and inflammation cells were evaluated by H&E staining (Fig. 3E). Rare fibroblasts and inflammatory cells could be seen in the NGCs within 2 weeks, suggesting the NGCs prevented the fibrous tissue ingrowth effectively and provoked mild acute inflammatory responses. At 4^th^ and 8^th^ weeks, the NGCs elicited a foreign body reaction, and fibrous tissue with dispersing neocapillaries could be observed in the NGCs.

### *In vitro* cell compatibility of cryoGelMA NGCs

**Figure 4:**
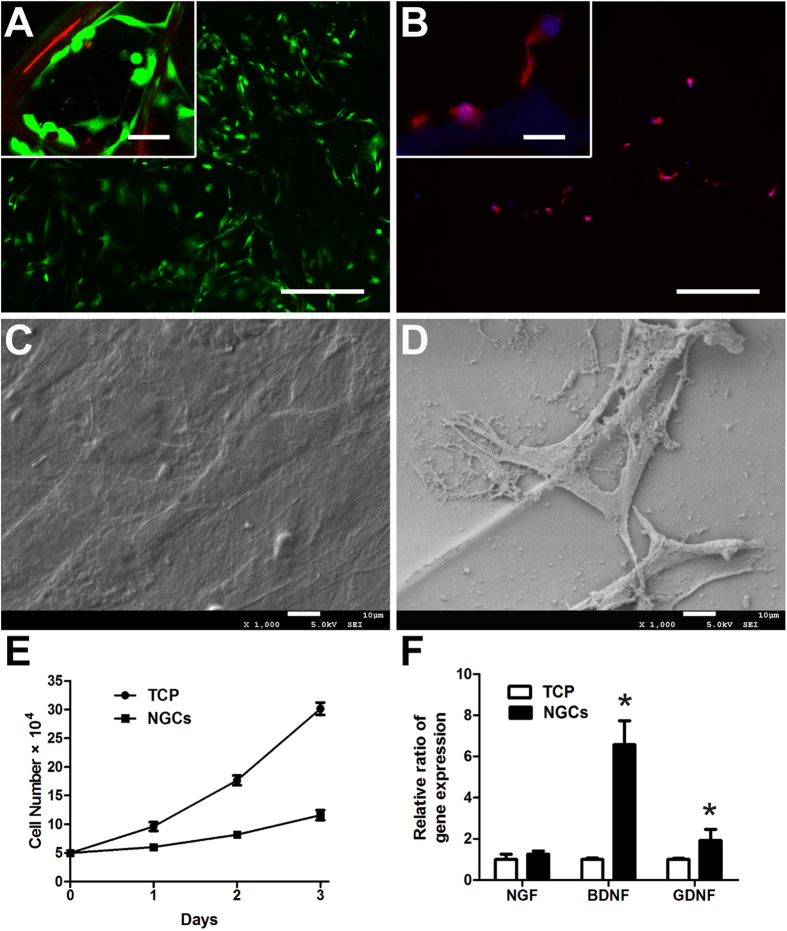
The attachment, proliferation, survival, and secretion of neurotrophic factors of ASCs on cryoGelMA NGCs *in vitro*. (**A**) Live/dead analysis of ASCs on the NGCs after 2 days of cell seeding (Scale bars = 200 μm, scale bars in upper left inset = 25 μm). (**B**) Staining of F-actin by rhodamine phalloidin. The ASCs spreaded on the surface of NGCs after 2 days of culture (Scale bars = 200 μm, scale bars in upper left inset = 25 μm). SEM micrographs of ASCs cultured on the TCP (**C**) and NGCs (**D**). (**E**) Analysis of the proliferation of ASCs on the TCP and NGCs after 1, 2, and 3 days of culture (n = 3). (**F**) Gene expression of major neurotrophic factors (NGF, BDNF, and GDNF) of ASCs on the TCP and NGCs at 2 days post-seeding. *p < 0.05 for comparison with the TCP.

The suitability of the cryoGelMA NGCs as a substrate for cell attachment and growth was further investigated. Rat ASCs were found distributed throughout the NGCs with rare dead cells as revealed by the live/dead staining (Fig. 4A). The ASCs attached to the surface of NGCs and maintained their fibroblast-like morphology after 2 days of culture (Fig. 4B,D). The deposition of ECMs by the ASCs was more apparent on the NGCs than that on the tissue culture polystyrene (TCP) (Fig. 4C,D). AlamarBlue assay was carried out to evaluate the cell viability, and a lower proliferation rate was observed on the NGCs (Fig. 4E). The gene expression of neurotrophic factors of the ASCs cultured on the NGCs and TCP was further tested. As shown in Fig. 4F, the expression of BDNF and GDNF was significantly up-regulated after cultured on the NGCs (p < 0.05).

### *In vivo* nerve regeneration

**Figure 5:**
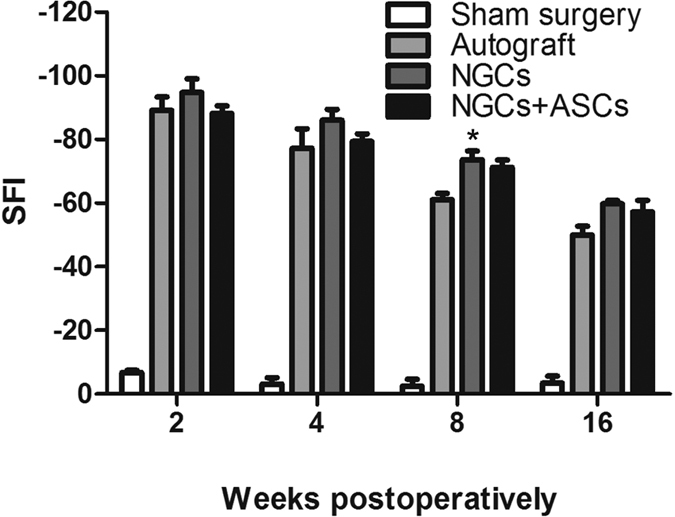
The SFI values of rats at 2, 4, 8 and 16 weeks after treatments (n = 4). *p < 0.05 for comparison with the autograft group.

The rat sciatic nerve transection model has been commonly used for the evaluation of tissue engineered NGCs in promoting peripheral nerve regeneration *in vivo*^28^. The cryoGelMA NGCs were implanted into the injured sciatic nerve to evaluate the nerve regeneration behaviors through the prepared four groups (sham surgery, autograft, NGCs, NGCs + ASCs). All rats were in good health condition throughout the experiment. To assess the functional recovery of the sciatic nerve, walking track analysis was performed on all operated animals at 2^nd^, 4^th^, 8^th^ and 16^th^ weeks post-surgery (Fig. 5). Sham surgery did not affect the SFI value significantly. The autograft group showed a significantly higher SFI value than the NGCs group at 8^th^ and 16^th^ weeks (p < 0.05), but there was no significant difference between the autograft group and the NGCs + ASCs group. Though repair with NGCs had a lower SFI value than that of NGCs+ASCs, no significant difference was observed.

**Figure 6:**
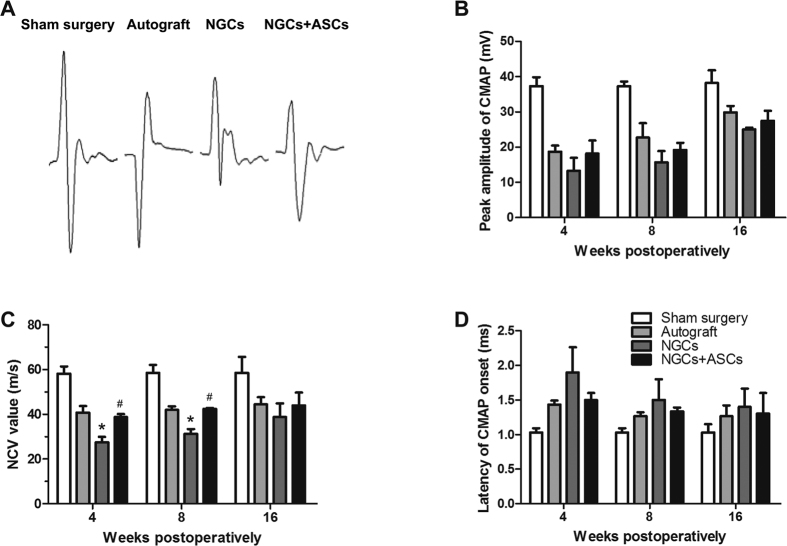
Electrophysiological assessments of the regenerated nerves in different treatment groups at 4, 8, and 16 weeks postoperatively. (**A**) Representative CMAP recordings at the injured side in each group at 16 weeks after implantation. The peak amplitude of CMAP (**B**), NCV value (**C**), and latency of CMAP onset (**D**) were recorded at different intervals after surgery. ^∗^p < 0.05 for comparison with autograft group, and ^#^p < 0.05 for comparison with NGCs group.

Electrophysiological analysis was performed at 4^th^, 8^th^, and 16^th^ weeks postoperatively (Fig. 6). CMAP has been commonly used to determine the numbers of regenerated motor nerve fibers^29^. The action potentials were observed in all groups at 4^th^ week and still intensive at 8^th^ and 16^th^ weeks after surgery, indicating a gradual enhancement of functional recovery for the injured nerves^30^,^31^. In consistence with the SFI findings, the CMAP in the NGCs + ASCs group was close to the autograft group, and there was no significant difference between the NGCs + ASCs and the NGCs group. NCV is an objective index that offers important insight for evaluating the conduction of action potentials in peripheral nerve^31^. The NCV value of the NGCs group was significantly lower than that of the autograft and NGCs + ASCs groups at 4^th^ and 8^th^ weeks after implantation (p < 0.05), but no statistically significant difference was observed at 16 weeks among the three groups. Latency of CMAP onset was decreased over time for the autograft, NGCs, and NGCs + ASCs groups, but no significant difference was observed among the three groups.

**Figure 7:**
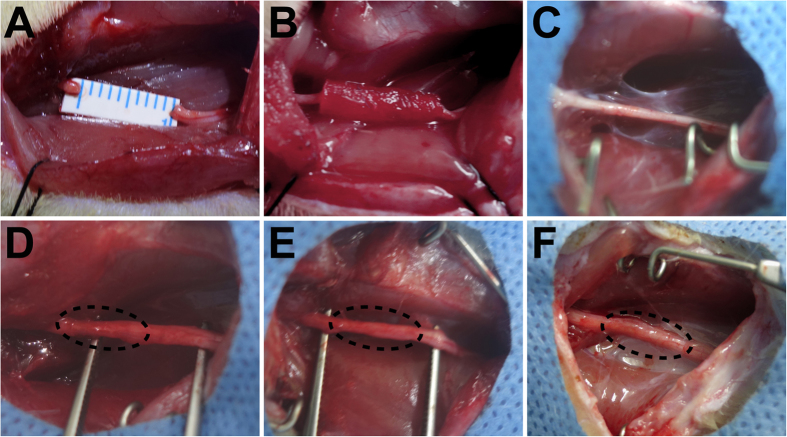
Intraoperative photographs of the cryoGelMA NGCs for nerve regeneration in a rat sciatic nerve transection model. (**A**,**B**) The rat sciatic nerve was transected to create a 10 mm gap and bridged with the 3D-printed NGCs. The general observations of the regenerated sciatic nerve in the sham surgery (**C**), autograft (**D**), NGCs (**E**), and NGCs + ASCs (**F**), groups at 16 weeks after surgery.

**Figure 8:**
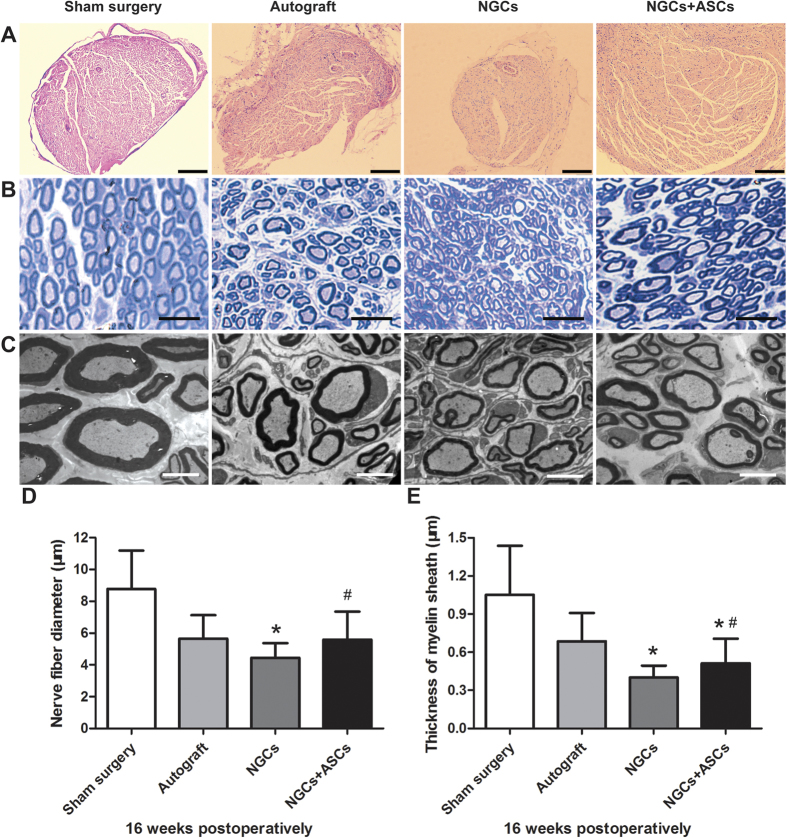
Histological assessment of the regenerated sciatic nerves in the distal segment at 16 weeks after surgery. (**A**) H&E staining shown the overview of nerve morphology in each group (Scale bars = 200 μm). Myelination of regenerated nerves revealed by toluidine blue staining (**B**, scale bars = 25 μm) and TEM (**C**, scale bars = 5 μm). Statistical analysis of the diameters of myelinated nerves (**D**) and thickness of myelin sheath (**E**) for each group (n = 4). ^∗^p < 0.05 for comparison with autograft group, and ^#^p < 0.05 for comparison with NGCs group.

Macroscopically, the cryoGelMA NGCs were all degraded completely, and the proximal and distal stumps were successfully reconnected by the regenerated nerve at 16^th^ week after surgery (Fig. 7). H&E staining showed the general regenerated nerve fibers morphology in the distal segment at 16^th^ week after implantation (Fig. 8A). The axon diameter and myelin sheath thickness of the distal segments of regenerated nerves were then evaluated by toluidine blue staining and transmission electron microscope (TEM) (Fig. 8B–E). The sham surgery group has the largest axon diameter and thickest myelin sheath. The axon diameter and myelin sheath thickness of the NGCs + ASCs group were significant longer than that of the NGCs group (p < 0.05). Although myelin sheath in the NGCs + ASCs group was thinner than that of the autograft group (p < 0.05), there was no significant difference in the axon diameter.

**Figure 9:**
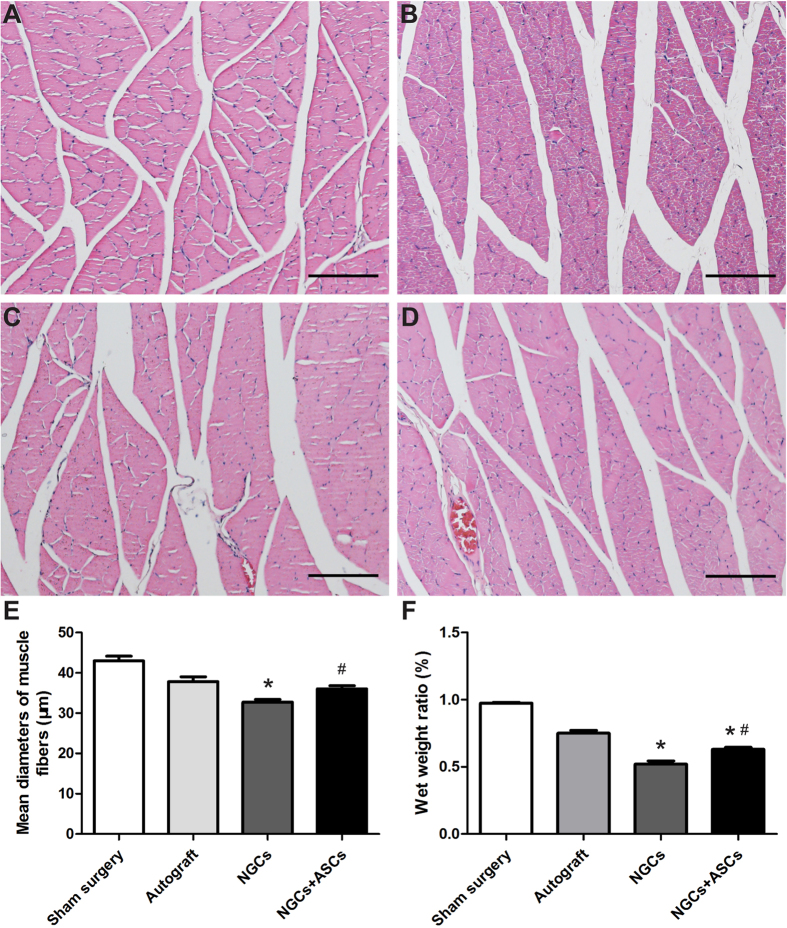
The gastrocnemius muscles atrophy and re-innervation. (**A**–**D**) H&E staining of gastrocnemius muscles cross-section in the sham surgery (**A**), autograft (**B**), NGCs (**C**), and NGCs + ASCs (**D**), groups at 16 weeks after implantation (Scale bars = 100 μm). (**E**) The mean diameters of muscle fibers and (**F**) the wet weight of the operated side to the non-operated side in different groups (n = 4). ^∗^p < 0.05 for comparison with autograft group, and ^#^p < 0.05 for comparison with NGCs group.

After sciatic nerve transection, the gastrocnemius muscles demonstrated degradations of muscle fibers and became fragments, and then regained nerve re-innervation^32^. H&E staining was performed to evaluate the recovery of nerve function in all groups (Fig. 9A–D). Compared to normal muscle morphology in the sham surgery group, gastrocnemius muscles were degenerated in the autograft and NGCs + ASCs groups, and prominently in the NGCs group. The muscle fiber diameter in the sham surgery, autograft, NGCs, and NGCs + ASCs groups was 43.0 ± 8.3 μm, 37.8 ± 8.6 μm, 33.9 ± 4.5 μm, and 36.0 ± 5.5 μm, respectively (Fig. 9E). The relative gastrocnemius muscle weight increase was similar to the increase in muscle fiber diameter in the four groups (Fig. 9F). The muscle fiber diameter and weight observed in the NGCs + ASCs group significantly increased when compared with that of the NGCs group.

## Discussion

In this work, we demonstrated a flexible method for the fabrication of cryoGelMA NGCs with desired geometries by a low cost desktop 3D printer. At present, the desktop 3D printer is being widely used in education, manufacturing, and industry for its low cost and versatility in fabrication^33^. With the help of the desktop 3D printer, rationally designed “lock and key” moulds were printed to fabricate NGCs with complex or personalized geometries for peripheral nerve regeneration. Using the “lock and key” moulds, the prepared NGCs could be acquired without dissolving the negative moulds by organic solvent, thus reducing the manufacturing time and avoiding organic solvent influences the internal structural characteristics of the conduits, such as pore size^34^,^35^. Conventionally, the NGCs are simple cylindrical structures, and it’s difficult and time-consuming to fabricate NGCs with complex architectures^18^,^19^,^36^. The fabrication of NGCs with advanced structures, such as multichannel, has been reported in previous studies using a wire mesh method^36^,^37^,^38^,^39^. However, the conventional method is infeasible to fabricate NGCs with versatile structure parameters, such as the internal diameter or wall thickness, that might affect the efficacy of nerve regeneration^5^,^6^,^40^. Take advantages of the indirect 3D printing technique, we can reliably fabricate NGCs with advanced structures and control the structure parameters. Our technique has advantages in terms of simplicity, flexibility and low cost in fabricating designer NGCs with complex or personalized geometries for peripheral nerve regeneration, which may lead to the development of future NGCs for clinical use.

Recently, microstereolithography was used for patterning NGCs with intricate geometries, and a 3D printing methodology was also applied to construct anatomical nerve regeneration pathways based on 3D scanning of a rat bifurcating nerve^14^,^18^,^19^. However, to our knowledge, the fabrication of a NGC based on patient-specific anatomy has never been explored. MR neurography has been increasingly used in recent years to evaluate abnormalities such as nerve injury, entrapment, and neoplasm^41^,^42^. The complex 3D anatomy of peripheral nerve is resolvable with multiplanar reconstruction, which provides prudently information of the length of injured nerve and the geometries of the proximal and distal discontinuous nerve stumps^2^. In this work, we performed an attempt to integrated 3D printing methodology with MR neurography to engineer a NGC based on the anatomic geometries of a patient without any aggressive manipulations, which allows the customization of a NGC that precisely match a particular nerve defects of a patient^18^.

Both *in vitro* and *in vivo* degradation studies show that the cryoGelMA NGCs were degradable. The NGCs have a proper degradation rate (completely degraded at 2–4 months), which is similar to several FDA approved NGCs, such as Neurotube^®^ and AxoGuard^TM^ Nerve Connector^6^. We found that ASCs cultured on the NGCs gave rise to more ECMs accumulation than that on the TCP. Furthermore, the expression of several neurotrophic factors mRNAs, such as BDNF, was unregulated after seeding the ASCs on the NGCs. The enhancement of cell-cell and cell-matrix interactions on the NGCs provides a more physiologically relevant environment to the ASCs, and thus enhances their functions^43^. Those results suggested that the introduction of ASCs into cryoGelMA NGCs may provide a favorable repair-conducive environment for nerve regeneration^26^. *In vivo* experiments also confirmed a benefit of applying the ASCs in promoting nerve regeneration after transplanted into the injured site. Several previous studies have demonstrated the utilization of ASCs improved axonal regeneration *in vivo*, and this effect has been attributed mainly to the environmental support provided by the ASCs during nerve regeneration^44^,^45^,^46^,^47^.

Walking track assessment and electrophysiological analysis are widely accepted techniques for the functional evaluation of sciatic nerve repair in rats^24^,^32^. We found that the cryoGelMA NGCs cellularized with ASCs showed similar results to the autograft group in function recovery and axonal regeneration as evidenced by no statistical difference in SFI, electrophysiological results, and nerve and muscle fiber diameters between the two groups. Although we did not observe significant beneficial effects on gait and electrophysiological analysis in ASCs-seeded NGCs compared to bare conduits, the significantly increased axonal regeneration and nerve re-innervation of gastrocnemius muscle suggested the application of ASCs promoted nerve regeneration in histomorphological parameters. The discrepancy between the outcomes concerning motion analysis and tissue structures is in line with several previous study^32^,^45^. A higher dose of ASCs may show improved functional recovery as a relatively low dose of ASCs (1 × 10^6^) was used in our study^46^,^48^.

## Conclusion

We described an indirect 3D-printing technology for fabricating cellularized designer cryoGelMA NGCs for peripheral nerve regeneration. By molding with 3D-printed “lock and key” moulds, NGCs with desired geometry, such as multichannel, bifurcating and the personalized structures, could be obtained by a low cost desktop 3D printer. The ASCs-cellularized NGCs were comparable with that of the autografts in repairing a peripheral nerve defect, showing potential clinical application in peripheral nerve regeneration.

## Materials and Methods

### Synthesis of GelMA

GelMA was synthesized following the procedures described elsewhere^13^. Briefly, gelatin type A (300 bloom from porcine skin, Sigma) at 10% (w/v) was dissolved into stirred Dulbecco’s phosphate buffered saline (DPBS) at 60 °C. Methacrylation of gelatin was achieved by adding 20% (v/v) of methacrylic anhydride (Sigma) at a rate of 0.5 mL/min and reacting at 50 °C for 1 h. Following a 5× dilution with warm DPBS (40 °C), the mixture was dialyzed against distilled water at 40 °C for 2 weeks. The sample was then lyophilized and stored at −20 °C until further use.

### CryoGelMA NGCs fabrication

CryoGelMA NGCs with desired structures, such as multichannel and bifurcating, were prepared by molding based on “lock and key” moulds that were designed *via* SolidWork version 2012 (SolidWorks Corp., Waltham, Massachusetts, USA) and manufactured by a commercially available desktop ink-jet printer (TD-IIA, TD ARTIST, Chengdu, China). To fabricate a cryoGelMA NGC based on anatomic geometry of a patient, the shape of sciatic nerve was isolated from the MR neurography by a commercially available software (Mimics, Materialise). Based on the image data, “lock and key” moulds were designed and converted into an STL file followed by 3D printing^49^. To further investigate the degradability, biocompatibility, and efficacy of nerve regeneration of the cryoGelMA NGCs, a 10 mm right sciatic nerve was acquired from the mid-thigh level of SD rats (220–250 g). The diameters of proximal and distal stumps of the injured nerve were measured for designing moulds.

GelMA prepolymer solution was formed by dissolving 5% (w/v) GelMA in dH2O and maintained at 60 °C to dissolve adequately. After incubated on ice for 5 min, 0.5% (w/v) ammonium persulfate (APS; Sigma) and 0.1% (w/v) tetramethylethylenediamine (TEMED; Sigma) were added to the prepolymer solution. The solution was then pipetted into the 3D-printed moulds cavity and underwent cryopolymerization for 24 h in a −20 °C refrigerator. The resulting cryogels were hydrated and harvested, and washed by deionized water extensively. After immersed in 75% ethyl alcohol for one hour, the gels were washed and collected into cell culture dish, lyophilized overnight (Boyikang), and stored in −20 °C prior to use.

### SEM

The morphology of the cryoGelMA NGCs were observed by using scanning electron microscopy (SEM, Hitachi S4800) operated at 5.0 kV. For this observation, the lyophilized conduits were mounted on aluminum stubs and gold-coated for 90 s before SEM imaging.

### Degradability

CryoGelMA NGCs were incubated with 1 mg/ml collagenase type II (Sigma) in DPBS and were placed on an orbital shaker at 100 rpm and 37 °C. At predefined time points (t = 4, 8, 12, 16, and 20 hours), the conduits were harvested. The degradation of the NGCs was calculated as a ratio of loss of dried weight to the primary weight. For *in vivo* degradation study, the conduits were implanted subcutaneously on the back of SD rats. At predefined time points (1, 2, 4 and 8 weeks) after implantation, the implants were photographed and harvested together with surrounding tissue for histological evaluation. We confirm that all experiments were performed in accordance with the guidelines and regulations of Sichuan University Committee on Animal Research and Ethics and were approved by the Institutional Animal Care and Use Committee of West China Hospital of Sichuan University.

### Cell adhesion and proliferation

Rat ASCs were isolated from subcutaneous adipose tissue of inguinal region of SD rats. ASCs were then maintained in Dulbeccos’ modified Eagles’ medium (DMEM, Invitrogen) with low glucose, containing 10% (v/v) fetal bovine serum (FBS, Invitrogen), 100 U/ml penicillin, 100 μg/ml streptomycin. Cells were trypsinized upon 80% confluency and each conduit was seeded with 1 × 10^5^ cells. 2 days after seeding, Live/Dead assay (Invitrogen) was performed for visualization of cell viability using a Zeiss confocal microscope (Zeiss). Moreover, F-actin was stained using rhodamine phalloidin (Cytoskeleton) and cell nucleus was stained using DAPI (Invitrogen), and images were taken on the confocal microscope. The morphology of ASCs on the cryoGelMA NGCs was visualized using SEM. After fixed with 3% glutaraldhyde for 2 h, specimens were rinsed with PBS and dehydrated with graded concentrations (30, 50, 70, 90, 100% v/v) of ethanol. Then the samples were coated with gold and observed with SEM.

Cell proliferation was determined by AlamarBlue assay (Invitrogen). Briefly, 5 × 10^4^ cells in 50 μl were seeded on each conduit and incubated for 1.5 h to allow for cell attachment. Then 1 ml fresh medium was added and the composites were cultured in 24-well plates for 24, 48 and 72 h. Meanwhile, ASCs of same density were seeded on the TCP as the control. After each time point of cell seeding, the culture medium was removed and 1 ml of 10% (v/v) Alamar blue in culture solution was added. After 3 h of incubation, aliquots were pipette into a 96-well plate and the absorbance at 570 nm and 595 nm was measured. Cell numbers were determined by a standard curve.

### RT-PCR

Total RNA was extracted using the Trizol^®^ reagent (Invitrogen) form ASCs cultured on conduits and TCP for 2 days. RNA strand was reverse transcribed into cDNA and amplified using PrimeScript II 1st Strand cDNA Synthesis Kit (TaKaRa). Polymerase chain reactions (PCRs) were performed using primers for glyceraldehyde 3-phosphate dehydrogenase (GAPDH), nerve growth factor (NGF), brain-derived neurotrophic factor (BDNF), and glial cell-derived neurotrophic factor (GDNF) as previously described^50^. PCRs were carried out with SYBR Premix Ex Taq (TaKaRa). The resulting amplification was monitored with the CFX96 Real-Time System (Bio-Rad). The expression levels were normalized against the reference gene GAPDH, and the relative gene expression was analyzed. The primer sequences for each gene used in this study are shown in Table S1.

### Animal and surgical procedure

SD rats weighing 220–250 g were used and randomly divided into four experimental groups: sham surgery group (n = 6), autograft group (n = 12), NGCs group (n = 12) and NGCs + ASCs group (n = 12). 1 × 10^6^ ASCs at early passages (P3–P6) were seeded on each conduit and cultured for 3 h at 37 °C to allow cells fully attached to the cryoGelMA scaffolds. After anesthetized by intraperitoneal chloral hydrate, the hair on the right femur was removed. Under aseptic conditions, the right sciatic nerve was exposed, and a 10 mm segment of the sciatic nerve was removed at the mid-thigh level. The NGCs were interposed between the proximal and distal nerve stumps. The nerve stumps were inserted into the NGCs to a depth of 2.5 mm, and fixed with 8-0 absorbable vicryl sutures. For autograft, the 10 mm transected nerve was re-implanted under microscope. Following the implantation, the muscle incision was closed using 5-0 vicryl sutures and the skin was closed with 2-0 silk sutures. Postoperatively, animals were free access to food and water, and housed in a controlled room with 12 h light cycles.

### Walking track analysis

The motor functional recovery was evaluated by walking track analysis at 2, 4, 8 and 16 weeks post-operatively. Pre-operatively, all rats were underwent conditioning training in a 50 cm × 8 cm wooden track. After the hind feet dipped in black ink, rats were allowed to walk down the track. Five measurable footprints were collected for each rat. Sciatic Function Index (SFI) was calculated by the formula proposed by Bain *et al*.^51^ as follows:


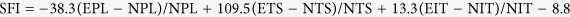


The print length (PL) is the distance from the heel to the third toe, the toe spread (TS) is the distance from the first to the fifth toe, and the intermediary toe spread (IT) is the distance from the second to the fourth toe. EPL, ETS, and EIT represent recordings from the operated foot, as well as NPL, NTS and NIT from the non-operated foot. A value of −100 implies total impairment.

### Electrophysiological analysis

Electrophysiological tests were performed using a previous developed method at 4, 8 and 16 weeks after implantation^30^. An electromyograph machine (Nuocheng, Shanghai, China) was used to measure all the experimental animals. After the sciatic nerve was exposed under anesthetization, a stimulating electrode was placed at the proximal side of regenerated nerve, and a recording electrode was inserted into the gastrocnemius muscle. The reference electrodes were positioned between the stimulating electrode and the recording electrode. The ground electrode was placed in the tail. For quantitative analysis, the peak amplitude of compound muscle action potential (CMAP), nerve conduction velocity (NCV), and latency of CMAP onset values were calculated respectively.

### Histological assessment

The distal nerve segments were harvested immediately after electrophysiological examination at 16 weeks after implantation. The paraformaldehyde-fixed nerves were dehydrated, embedded in olefin, cut into 5 μm-thick slices, and stained with hematoxylin/eosin (H&E). The other samples were embedded in Epon 812 epoxy resin and stained with toluidine blue after cut into semi-thin sections (0.5 μm). The images of histological sections were captured and analyzed using a digital image analysis system (Nikon E600 Microscope with a Nikon Digital Camera DXM 1200, Nikon Corporation, Japan). To observe the ultrastructure of myelin sheath, ultrathin sections (50 nm) were viewed and photographed with a Hitachi H7650 TEM (Tokyo, Japan). The diameters of myelinated axons and thickness of myelin sheath were quantified from TEM images using the Image J software. For each specimen, a total of 50–60 random axons were analyzed.

The gastrocnemius of both limbs was harvested from the bone attachments at 16 weeks after surgery. The moist weights of the gastrocnemius muscle were weighed on an analytic scale, and the ratio of wet weight on the experimental to contralateral sides in each group was calculated to evaluate the target muscle reinnervation. Then the middle portion of the muscle was dissected and placed in 4% paraformaldehyde for H&E staining. The diameters of muscle fibers were calculated from 5 random fields using the Image J software.

### Statistical analysis

Values are expressed as means ± standard deviation. Significant differences among groups were analyzed by single-factor analysis of variance (ANOVA) followed by Bonferroni’s post-hoc test using SPSS 16.00 software. Statistically significant differences between medians were determined with a Mann-Whitney U test. A value of p < 0.05 was considered statistically significant.

## Additional Information

**How to cite this article**: Hu, Y. *et al*. 3D-engineering of Cellularized Conduits for Peripheral Nerve Regeneration. *Sci. Rep*. **6**, 32184; doi: 10.1038/srep32184 (2016).

## Supplementary Material

Supplementary Information
