# Supplementary material for: 3D-engineering of Cellularized Conduits for Peripheral Nerve Regeneration
Source: Sci Rep. 2016 Aug 30;6:32184. doi: 10.1038/srep32184 (PMC5004136; doi:10.1038/srep32184)
Supplement: Supplementary Information [file srep32184-s1.pdf]

3D-engineering of Cellularized Conduits for Peripheral Nerve Regeneration

Yu Hu†,‡,#, Yao Wu‡,#, Zhiyuan Gou†,#, Jie Tao†, Jiumeng Zhang†, Qianqi Liu†, Tianyi Kang†,

Shu Jiang‡, Siqing Huang‡, Jiankang He §, Shaochen Chen∇, Yanan Du∮ , Maling Gou*,†

†State Key Laboratory of Biotherapy and Cancer Center, West China Hospital, Sichuan University, and Collaborative Innovation Center for Biotherapy, Chengdu, Sichuan province, China

‡Department of Neurosurgery, West China Hospital, Sichuan University, Chengdu, Sichuan province, China

§Xi'an JiaoTong University, School of Mechanical Engineering, Xi'an, Shanxi Province,China

∇ Department of NanoEngineering, University of California, San Diego, La Jolla, California 92093, USA

∮ Department of Biomedical Engineering, School of Medicine, Tsinghua University, Beijing 100084 , China

*Corresponding author: Maling Gou; email: goumaling@scu.edu.cn

#These authors contributed equally to this work and are co-first authors.

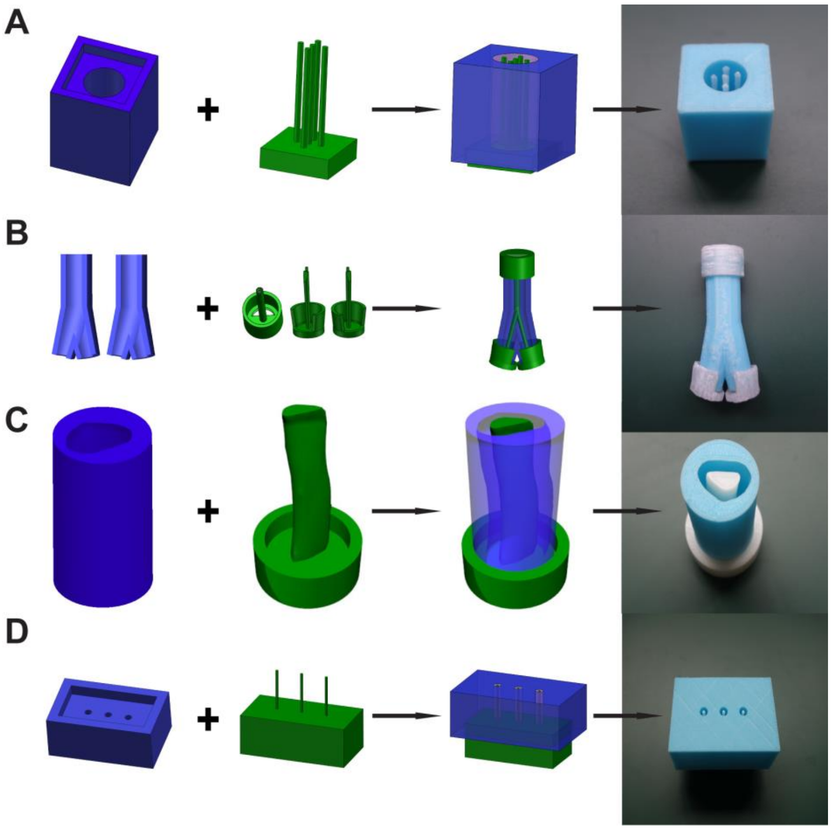

Figure S1. 3D-printed molds used for fabricating multichannel (A), bifurcate (B), patient-specific (C), and cylindrical (D) nerve guidance conduits.

Table S1. Primer sequences used for PCR amplification.

Genes	Primer sequences
GAPDH	Forward: CAGAACATCATCCCTGCATC
	Reverse: ACTCCTCAGCAACTGAGGG
NGF	Forward: CCAAATCCTTGGATTATCTGCTG
	Reverse: AAGCCTCTACTTATCCACCCAGG
BDNF	Forward: TGGCCTAACAATGTTTGCAGAT
	Reverse: CCACTCAGAAATTCCTCCTGCT
GDNF	Forward: TGGGCTATGAAACCAAGGAG
	Reverse: ATACATCCACACCGTTTAGCG
